# Experimental Study on Mechanical Properties and Failure Laws of Granite with Artificial Flaws under Coupled Static and Dynamic Loads

**DOI:** 10.3390/ma15176105

**Published:** 2022-09-02

**Authors:** Guang Li, Shuaiqi Liu, Rong Lu, Fengshan Ma, Jie Guo

**Affiliations:** 1 Key Laboratory of Shale Gas and Geoengineering, Institute of Geology and Geophysics, Chinese Academy of Sciences, Beijing 100029, China; 2 Institutions of Earth Science, Chinese Academy of Sciences, Beijing 100029, China; 3 Economics & Technology Research Institute, China National Petroleum Corporation, Beijing 100011, China; 4 Beijing Urban Construction Design & Development Group Co., Ltd., Beijing 100029, China

**Keywords:** coupled static and dynamic loads, artificial flaw, SHPB, 3D-DIC, failure laws

## Abstract

Rock is the main construction material of rock engineering, such as the engineering of mines and tunnels; in addition, its mechanical properties and failure laws are of great significance to the stability evaluation of rock engineering, especially under the conditions of coupled static–static stresses. In this study, granite specimens were manufactured with artificial flaws. Coupled static and dynamic loads tests were carried out with a modified split Hopkinson pressure bar (SHPB) apparatus; and six typical levels of axial pre-stresses and three crack inclination angles were designed. Three-dimensional digital image correlation (3D-DIC) was also applied to record and analyze the fracturing process and damage evolution of the specimens. The test results show that there was no compaction stage in the stress–strain curve under combined dynamic and static loading. The dynamic strength of the specimens increased first and then decreased with the increase in the static pressure; moreover, the specimens reached the maximum dynamic strength when the static pressure was 10% UCS. The dynamic strength decreased first and then increased with the increase in the crack inclination angle; and the lowest strength appeared when the inclination angle was 45°. The change in axial compression had a significant influence on the failure mode, and the failure mode gradually transformed from shear–tensile failure to shear failure with the increase in the pre-stress. The tensile strain was usually generated at the end of the fractures or near the rock bridge. When the axial pressure was small, the tensile strain zone parallel to the loading direction was easily generated; and when the axial pressure was large, a shear strain zone developed, extending along the diagonal direction. The research results can provide a theoretical reference for the correct understanding of the failure mechanisms of granite and its engineering stability under actual conditions.

## 1. Introduction

In the fields of resource exploitation, transportation, and underground engineering, the construction of deep rock engineering is more and more frequent; in addition, the research of deep rock mass mechanics has received extensive attention. Due to the influence of in situ stress, geological structure, weathering, human activities, and other factors, there are many defects in rock that weaken its ultimate bearing capacity; this includes cracks, holes, joints, foliation, faults, and so on [1,2]. Rock fractures usually start at the tip of the original defect, extend through each other, and finally form a whole failure [3,4]. In addition to the high ground stress, in the process of deep engineering construction, rocks experience different disturbances and are often subjected to strong dynamic loads; such as blasting vibration, excavation, drilling, etc., which is a combination of dynamic and static loads [5,6]. Both rock fractures and engineering disturbances are important factors that affect the mechanical properties of a deep high-stress rock mass. Moreover, deep hard rock has significant brittleness; this is more likely to cause engineering disasters, such as rock burst and rock engineering instability. Therefore, it is necessary to study the mechanical properties and failure behavior of defective rocks under combined dynamic and static loading.

Laboratory testing of rock fracture and damage under combined dynamic and static loading is an important method to understand this problem. Based on the improved split Hopkinson pressure bar (SHPB), Li et al. conducted impact experiments on marble with a single fracture; the results showed that the dynamic failure mode of a fractured rock mass was shear failure [7]. Wang et al. carried out one-dimensional dynamic and static combined loading tests on coal samples with different moisture content [8]. Li et al. studied the mechanical and failure characteristics of sandstone samples with prefabricated holes under dynamic–static loading [9]. Weng et al. reproduced the crack propagation and failure process of granite with round or square holes [10]. Li et al. conducted one-dimensional dynamic and static combined loading tests on sandstone samples with prefabricated holes to study the cracking behavior and its mechanism [11]. Yin et al. explored the influence of heat treatment temperature and loading rate on the dynamic fracture behavior of Brazilian disc granite containing fractures under a pre-static load [12]. In addition, some scholars have used the acoustic emission, speckle, and mathematical programming methods to explore the strength, fracture mechanism, and failure evolution law of defective rocks [13,14,15,16]. However, the above researchers have mostly selected relatively low-strength rock samples for the tests; moreover, the studies on the mechanical properties of deep hard rock are still insufficient. Meanwhile, there have been few studies on the role of static confining pressure and the inclination angle of fracture in the process of rock failure under coupled static and dynamic loads.

Therefore, a series of laboratory tests were designed and conducted to study the mechanical and failure characteristics of fractured rock under the combined dynamic and static loading; based on an improved SHPB system and digital image correlation (DIC) technology. These provide a theoretical reference for understanding the failure mechanism of granite and evaluating the stability of engineering surrounding rock.

## 2. Coupled Static and Dynamic Loading Tests

### 2.1. Creating the Specimens

**Figure 1:**
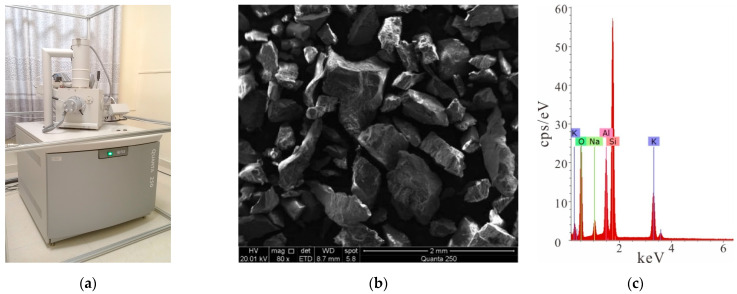
Microscopic scanning of the rock sample: (**a**) a picture of the scanner; (**b**) the microscopic scanning of the rock sample; (**c**) the element content characteristics of the rock sample.

**Figure 2:**
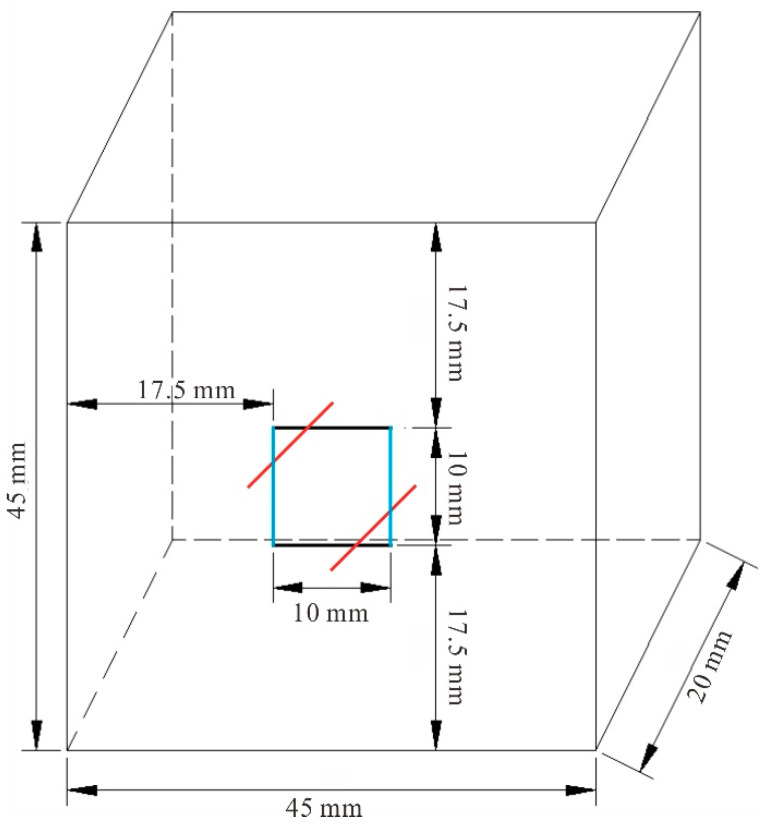
Specimen size.

Rock samples in the following tests were all taken from the Sanshandao gold mine, China. Granite with good integrity and homogeneity was used; with a density of 2580 kg/m^3^; an elastic modulus of 9.86 GPa; and a uniaxial compressive strength (UCS) of 139.66 MPa. A series of core slices were identified using optical microscopy. The rock sample composition is approximately 34% plagioclase, 27% potassium feldspar, 30% granular quartz, and 6% flaky biotite; and accessory minerals, such as calcite, white mica, and brown epidote, and so on, as shown in Figure 1 [17,18]. Rock cores were processed into cuboid samples, with a size of 45 mm × 45 mm × 20 mm (length × height × thickness), were divided into complete samples and crack samples with double parallel joints. A cutting machine was used to precast cracks in the granite specimens. The length of the fractures was 10 mm and the width was 1 mm, as shown in Figure 2. The specimens(University of South China, Hengyang, China) with cracks included three different inclination angles, which were 0°, 45°, and 90°, respectively.

### 2.2. Testing Equipment

**Figure 3:**
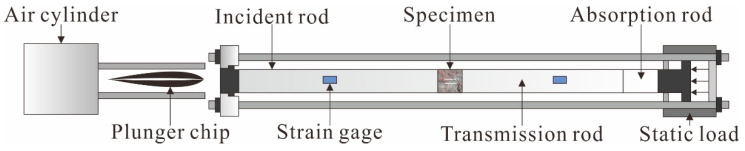
Testing equipment.

In this experiment, the SHPB device(Central South University, Changsha, China) was used to simulate the mechanical behavior of the specimens; with parallel double cracks under simultaneous dynamic and static loading. The SHPB device is mainly composed of three parts, namely the power drive system; the pressure rod system; and the data collection system, as shown in Figure 3 [19]. The pressure rod system includes an incident rod, a transmission rod, and an absorption rod. The specimen was placed between the incident rod and the transmitted rod; and the impact process was realized using air pressure to accelerate the warhead. Two sets of strain gauges were attached to the incident and transmitted rods to record the strain. In order to achieve the purpose of the constant strain rate loading, the spindle punch was used to realize the half-sine wave loading. Meanwhile, the system was equipped with an ultra-dynamic strain gauge and oscilloscope; this can realize the acquisition and record the stress wave signal and data processing. Based on the one-dimensional stress wave theory, the average dynamic stress, strain, and strain rate of the sample can be calculated by the following formula [20]:

$$\sigma \left(t\right)=\frac{A_{e}}{2A_{s}}\left[\sigma_{I}\left(t\right)-\sigma_{R}\left(t\right)+\sigma_{T}\left(t\right)\right]$$

$$\varepsilon \left(t\right)=\frac{1}{\rho_{e}C_{e}L_{s}}\int_{0}^{t}\left[\sigma_{I}\left(t\right)+\sigma_{R}\left(t\right)-\sigma_{T}\left(t\right)\right]d_{t}$$

$$\dot{\varepsilon }\left(t\right)=\frac{1}{\rho_{e}C_{e}L_{s}}\left[\sigma_{I}\left(t\right)+\sigma_{R}\left(t\right)-\sigma_{T}\left(t\right)\right]$$

where *σ_I_(t)*, *σ_R_(t)*, and *σ_T_(t)* are the incident stress, reflected stress, and transmitted stress of the rod at time *t*; *A_e_*, *ρ_e_*, and *C_e_* are the cross-sectional area, density, and longitudinal wave velocity of the rod; and *A_s_* and *L_s_* are the cross-sectional area and length of the sample, respectively.

DIC was used to observe the failure process of the rock under combined dynamic and static loading; which can measure the deformation process of the samples without direct contact, compared to other deformation measurement methods [21]. During the test, two high-intensity light-emitting diode (LED) lights were used to fill the light, two high-speed cameras were used to shoot, and DIC dynamic high-speed cameras(CSI Technology Group, West Hartford, CT, USA) were used to record the progressive failure process. After the artificial speckles were sprayed on the sample surface, the three-dimensional coordinates of the measured points on the object surface before and after the deformation could be obtained based on the principle of binocular stereo vision. Based on a series of continuous speckle images collected from different perspectives, the correlation matching operation was performed on all the markers of the two images before and after the deformation; in addition, the 3D displacement field and strain field on the surface of the sample during loading were finally obtained.

### 2.3. Experimental Project

The pre-loaded axial static pressure rates of the sample were set as 14.0, 27.9, 41.9, 69.8, and 83.8 MPa; these corresponded to 0%, 10%, 20%, 30%, 50%, and 60% of the UCS, and are represented by *S_0_*, *S_A_*, *S_B_*, *S_C_*, *S_D_*, and *S_E_*, respectively. The sample containing prefabricated cracks was referred to as ‘Flaw’. In other words, sample *S_B_*-flaw 45° represents a rock specimen containing 45° cracks with a pre-loaded axial static pressure of 10% UCS.

**Figure 4:**
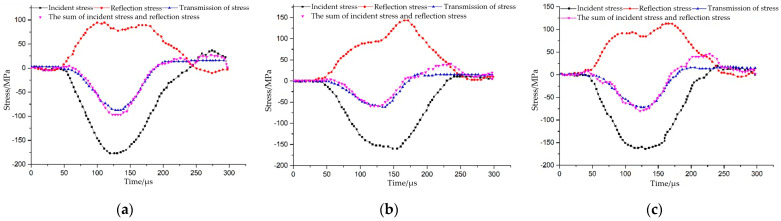
Stress equilibrium curves of the *S_B_*-flaw specimens: (**a**) *S_B_*-flaw 0°; (**b**) *S_B_*-flaw 45°; and (**c**) *S_B_*-flaw 90°.

In order to ensure the correctness of the SHPB test results, dynamic stress balance had to be reached at both sides of the sample before rock failure under the dynamic load. The stress equilibrium curves of the *S_B_*-flaw specimens were taken as examples. As shown in Figure 4, the superposition wave of the incident wave and reflected wave was basically consistent with the transmitted wave, especially the section before the peak; this indicates that the rock sample reached the stress equilibrium state during the loading period. In the deformation process of the rock samples, the assumption of the stress balance condition was satisfied; moreover, the improved SHPB device was consistent with the one-dimensional stress wave transfer characteristics, which can effectively eliminate the wave dispersion and inertia effects. Thus, this proves the validity of the test results.

## 3. Results and Discussion

### 3.1. Test Results

The test results of the dynamic and static combined loading are shown in Table 1; where the dynamic strength is the peak stress of the dynamic stress–strain curve, reflecting the impact resistance of the sample. The combined strength was the sum of the axial pre-static load and dynamic strength, reflecting the actual peak strength when the sample was damaged [22,23].

### 3.2. Deformation Characteristics

**Figure 5:**
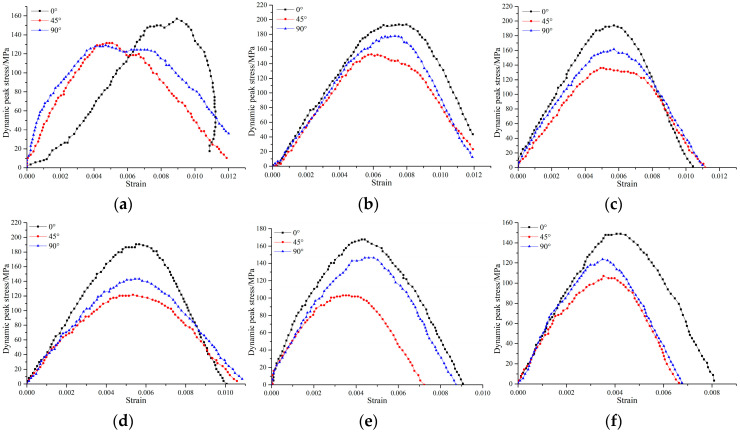
Dynamic stress–strain curves of different inclination angles under static axial compression: (**a**) 0 MPa; (**b**) 10% UCS; (**c**) 20% UCS; (**d**) 30% UCS; (**e**) 50% UCS; and (**f**) 60% UCS.

The stress–strain relationship of the sample was obtained from the signal transformation collected by the strain gauge, as shown in Figure 5a–f. Overall, different from general static load stress–strain curves, the stress–strain curves under dynamic and static loading did not have a compaction phase, and began directly at the elastic stage. This is because the pre-loaded static pressure had a compaction effect on the sample before the dynamic load loading, and the micro defects inside the sample were closed. The stress–strain curve of the combined dynamic and static loading can be divided into the pre-failure zone and post-failure zone; the stress peak point being the boundary. The pre-failure zone consisted of two stages, namely the elastic stage and the yield stage. Due to the fast loading rate, the rock samples quickly reached the peak strength and then completely failed; in addition, there was nearly no strain-softening stage or residual strength stage.

The results are shown in Table 1 and Figure 5. When the crack angle was constant and the axial static pressure increased gradually in the range of 0~83.8 MPa (0%~60% UCS), the dynamic strength of the specimen rose first and then declined. When the static pressure was 10% UCS, the dynamic strength of the specimens reached the maximum. The pre-stress played the role of compaction on the specimen and increased its ability to resist the dynamic force. However, with the increase in the pressure value, the static pressure itself caused damage to the specimen; thus, reducing the strength of the specimen. Under the same static pressure, the dynamic strength of the sample first decreased; it then increased with the increase in the crack inclination angle. The strength was weakest as the inclination angle of the fissure was 45°. Therefore, in actual engineering, more attention should be paid to the change trend of the fissure dip angle in the surrounding rock; this is conducive to our judgment on engineering stability.

### 3.3. Failure Modes

**Figure 6:**
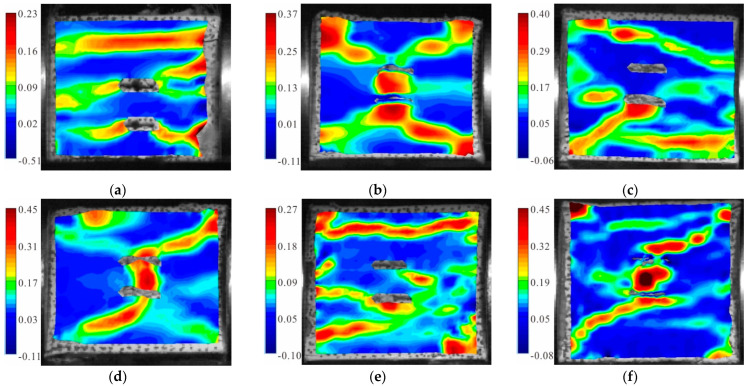
Maximum principal strain cloud diagram of the granite specimens with 0° cracks: (**a**) S_0_-flaw 0°; (**b**) S_A_-flaw 0°; (**c**) S_B_-flaw 0°; (**d**) S_C_-flaw 0°; (**e**) S_D_-flaw 0°; and (**f**) S_E_-flaw 0°.

Figure 6 shows the maximum principal strain cloud diagram of the fractured granite specimens with a 0° dip angle under different axial pressures. It can be seen from the figure that the axial compression significantly affected the failure mode of the sample. When the static load was 0, three tensile strain zones parallel to the loading direction were generated in the specimen, two of which ran through the cracks; the final result was obvious tensile failure (Figure 6a). When the static load was 10% UCS, two inclined tensile strain bands led to the sample failure; in addition, a large tensile strain zone was generated between the parallel double cracks (Figure 6b). When the static load was 20% UCS, an inclined shear strain zone through the sample appeared in the upper part of the specimen; and a compound strain zone with shear strain and tensile strain developed around the lower crack (Figure 6c). When the static load was 30% UCS, a set of “X”-shaped shear strain zones centered on the fracture developed in the specimen; moreover, two parallel fractures were connected due to the shear strain (Figure 6d). When the static load was 50% UCS, the upper part of the specimen generated a penetrated tensile strain zone parallel to the loading direction; the lower part formed a composite zone of tensile and shear strain through the cracks; and a large local tensile strain failure occurred on the lower right corner (Figure 6e). When the static load was 60% UCS, the failure of the specimen was caused by a primary shear strain zone connecting the lower crack; and a secondary shear strain zone connecting the upper crack of the specimen. In addition, a large tensile strain zone was generated between the parallel cracks and the upper left part of the specimen (Figure 6f).

**Figure 7:**
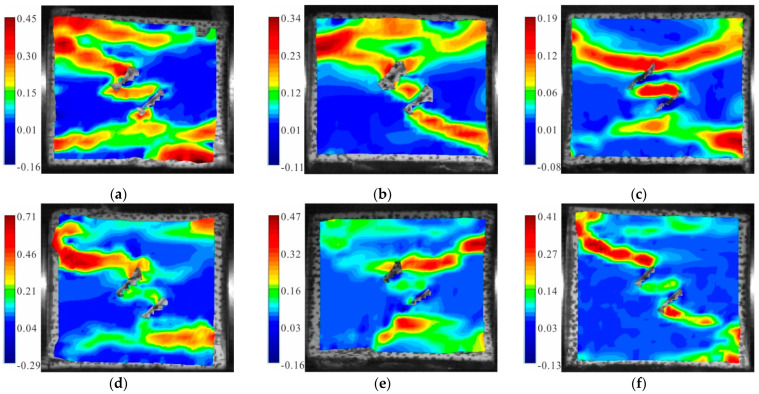
Maximum principal strain cloud diagram of the granite specimens with 45° cracks: (**a**) S_0_-flaw 45°; (**b**) S_A_-flaw 45°; (**c**) S_B_-flaw 45°; (**d**) S_C_-flaw 45°; (**e**) S_D_-flaw 45°; and (**f**) S_E_-flaw 45°.

Figure 7 shows the maximum principal strain cloud diagram of the fractured granite specimens with a 45° dip angle under different axial pressures. It can be seen from the figure that anti-wing shear strain zones always developed in the sample; and tensile strain zones formed at the tip of the parallel double fracture and were parallel to the loading direction. When the static load was 0, two tensile strain concentration zones developed on the upper left corner and the lower right corner of the specimen, respectively; they connected with the counter-wing shear strain zone, resulting in the failure (Figure 7a). When the static load was 10% UCS, an inclined shear strain zone from the upper left to the lower right and a horizontal tensile strain zone formed in the specimen; in addition, a violent deformation failure occurred between the two strain concentration zones (Figure 7b). When the static load was 20% UCS, a horizontal tensile strain zone connected to the fracture tip formed in the upper and lower parts of the specimen, respectively; together, these cracks led to a shear–tensile failure (Figure 7c). When the static load was 30% UCS, except for an anti-wing shear strain zone along the diagonal through the specimen, a small shear strain zone also appeared along the fracture. The two strain concentration zones connected with each other and extended to the boundary of the specimen to cause damage (Figure 7d). When the static load was 50% UCS, there was a small shear strain zone at the upper crack tip and a tensile strain zone at the lower crack tip; together, they caused the sample failure (Figure 7e). When the static load was 60% UCS, a typical anti-wing shear strain zone along the diagonal direction formed in the specimen; while a horizontal tensile strain region also appeared in the upper part (Figure 7f).

**Figure 8:**
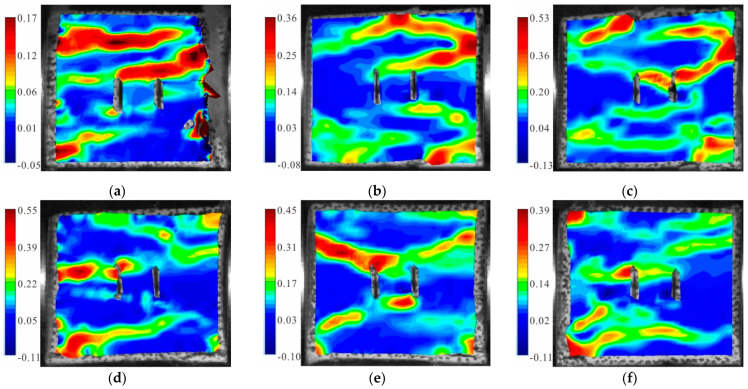
Maximum principal strain cloud diagram of the granite specimens with 90° cracks: (**a**) S_0_-flaw 90°; (**b**) S_A_-flaw 90°; (**c**) S_B_-flaw 90°; (**d**) S_C_-flaw 90°; (**e**) S_D_-flaw 90°; and (**f**) S_E_-flaw 90°.

Figure 8 shows the maximum principal strain cloud diagram of the fractured granite specimens with a 90° dip angle under different axial pressures. It can be seen from the Figure that when the static load was 0, there were two main tensile strain zones and one secondary tensile strain zone in the specimen. One of the main tensile strain zones ran through the tip of the double fractures, and the expansion of the three strain concentration zones jointly led to the final failure (Figure 8a). When the static load was 10% UCS, a tensile crack through the cracks was generated in the upper part of the specimen and connected with the upper tensile strain zone at the right boundary of the specimen; while a shear strain zone was generated near the lower part of the cracks (Figure 8b). When the static load was 20% UCS, there were two tensile strain zones in the lower part of the specimen: one of which was approximately parallel to the loading direction and went through the specimen; while the other one developed through the lower tip of the cracks. Meanwhile, a shear strain zone developed in the upper part and along the inclined direction (Figure 8c). When the static load was 30% UCS, two shear strain zones were generated in the upper left and lower left of the specimen, respectively; both of which tended to develop toward the fracture tip. In addition, a principal tensile strain zone was formed between the upper tip of the left fracture and the left boundary (Figure 8d). When the static load was 50% UCS, the sample failure was induced by two shear strain zones connecting the upper and lower tips of the double fracture, respectively (Figure 8e). When the static load was 60% UCS, a tensile strain zone formed in the lower part of the specimen; and a shear strain zone developed from two sides of the specimen to the cracks. These two strain concentration zones connected with each other and eventually lead to the failure (Figure 8f).

## 4. Conclusions

1. The dynamic strength of the specimens increased first and then decreased with an increase in static pressure; in addition, the specimen reached the maximum dynamic strength when the static pressure was 10% UCS. The pre-stress played the role of compaction on the specimen and increased its ability to resist the dynamic force. However, with the increase in the pressure value, the static pressure itself caused damage to the specimen; thus, reducing the strength of the specimen. The dynamic strength decreased first and then increased with the growth of the crack inclination angle; moreover, the lowest strength appeared when the inclination angle was 45°. The angle of 45° was conducive to the generation of shear strain zones along the diagonal direction, which often led to the final failure of the specimen.
2. The change in the axial compression had a significant influence on the failure mode. In terms of the specimen with 0° cracks, the specimen exhibited tensile failure in the absence of axial compression. Under uniaxial compression of 20% and 50%, the typical tensile shear failure occurred; and when the uniaxial compression was 10%, 30%, or 60%, the shear failure was predominant. The failure mode of the specimens with a 45° fracture was mainly a counter-wing shear strain developing around the end of the fractures; in addition, this shear mode became more and more obvious with the increase in the axial compression. The failure mode gradually transformed from shear–tensile failure to shear failure. The failure mode of the specimens with a 90° crack was mainly characterized by the tensile strain at the tips of the fracture. With the increase in the axial compression, the length and number of shear cracks increased; and the failure mode transformed from tensile failure to shear–tensile failure.
3. The inclination angle of the double parallel cracks played a key role in the formation and development of the tensile strain zone. The tensile strain was usually generated at the end of the fractures or near the rock bridge. When the axial pressure was small, the tensile strain zone parallel to the loading direction was easily generated; moreover, when the axial pressure was large, the shear strain zone extending along the diagonal direction developed.

## Figures and Tables

**Table 1:** Dynamic–static loading test results.

Sample	Static Pressure/MPa	Dynamic Strength/MPa	Combined Strength/MP	Peak Strain/10^−3^	Strain Rate/s^−1^
S_0_-flaw 0°	0	155.60	155.60	8.83	109.16
S_0_-flaw 45°		132.36	132.36	5.02	105.55
S_0_-flaw 90°		141.32	141.32	6.07	136.89
S_A_-flaw 0°	10% UCS	196.50	210.50	6.62	98.37
S_A_-flaw 45°		152.72	166.72	5.70	135.10
S_A_-flaw 90°		177.50	191.50	7.01	96.38
S_B_-flaw 0°	20% UCS	194.15	222.05	5.28	113.72
S_B_-flaw 45°		132.27	160.17	5.46	144.60
S_B_-flaw 90°		159.76	187.66	5.11	119.33
S_C_-flaw 0°	30% UCS	189.45	231.35	5.53	114.61
S_C_-flaw 45°		132.68	174.58	5.37	154.26
S_C_-flaw 90°		152.70	194.60	5.01	143.12
S_D_-flaw 0°	50% UCS	166.90	236.70	4.28	140.65
S_D_-flaw 45°		90.96	160.76	3.21	141.67
S_D_-flaw 90°		133.18	202.98	4.18	103.60
S_E_-flaw 0°	60% UCS	149.32	233.12	4.04	131.82
S_E_-flaw 45°		106.25	190.05	3.57	162.34
S_E_-flaw 90°		122.97	206.77	3.41	144.48

## Data Availability

Not applicable.
